# Unraveling Thermal Transport Properties of MoTe_2_ Thin Films Using the Optothermal Raman Technique

**DOI:** 10.1021/acsami.3c06134

**Published:** 2023-07-12

**Authors:** Carlos Rodriguez-Fernandez, Arttu Nieminen, Faisal Ahmed, Jesse Pietila, Harri Lipsanen, Humeyra Caglayan

**Affiliations:** † Faculty of Engineering and Natural Sciences, Photonics, Tampere University, 33720 Tampere, Finland; ‡ Department of Electronics and Nanoengineering, Aalto University, P.O. Box 13500, FI-00076 Aalto, Finland

**Keywords:** optothermal characterization, MoTe_2_, Raman spectroscopy, TMDs, thermal conductivity, and interfacial thermal conductance.

## Abstract

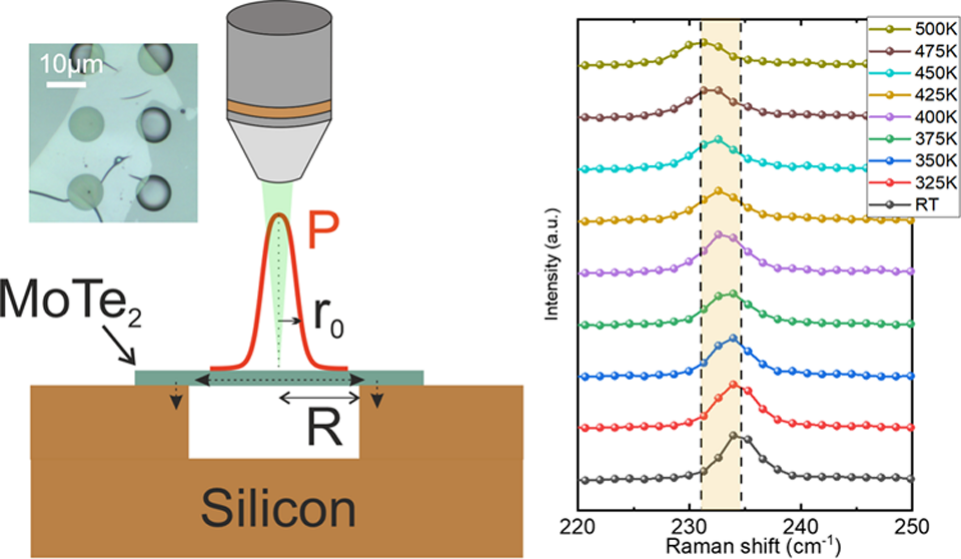

Understanding phonon transport and thermal conductivity of layered materials is not only critical for thermal management and thermoelectric energy conversion but also essential for developing future optoelectronic devices. Optothermal Raman characterization has been a key method to identify the properties of layered materials, especially transition-metal dichalcogenides. This work investigates the thermal properties of suspended and supported MoTe_2_ thin films using the optothermal Raman technique. We also report the investigation of the interfacial thermal conductance between the MoTe_2_ crystal and the silicon substrate. To extract the thermal conductivity of the samples, temperature- and power-dependent measurements of the in-plane E_2g_^1^ and out-of-plane A_1g_ optical phonon modes were performed. The results show remarkably low in-plane thermal conductivities at room temperature, at around 5.16 ± 0.24 W/m·K and 3.72 ± 0.26 W/m·K for the E_2g_^1^ and the A_1g_ modes, respectively, for the 17 nm thick sample. These results provide valuable input for the design of electronic and thermal MoTe_2_-based devices where thermal management is vital.

## Introduction

Transition-metal dichalcogenides (TMDs) have been extensively studied as potential materials for the next generation of optoelectronic devices because of their unique and tunable physical and chemical properties at nanometric scale, including thickness-dependent band gaps, strain engineering, high carrier mobility, broadband optical absorption, high optical response, etc.^1−7^ These layered materials exhibit strong covalent bonds in-plane and weak out-of-plane van der Waals forces that enable the preparation of uniform atomic layers by mechanical or chemical exfoliation. Moreover, these materials have good mechanical strength^8^ and nanoscale dimensionality, resulting in huge potential for electronic applications, energy, storage, biomedicine, catalysis, etc.^9−11^ Besides these applications, mono- and few-layer TMD materials have been considered forthcoming candidates for thermoelectric (TE) and energy-efficient device applications.^12,13^ The conversion efficiency to transform waste heat into electrical power of a TE material is defined by the dimensionless figure of merit ZT which is directly proportional to the square of the Seebeck coefficient and inversely proportional to the thermal conductivity.^14^ The dichalcogenides of group VI, 2H-MX_2_ (M = Mo, W; X = S, Se, Te) are promising materials for these applications, owing to their phonon properties, relatively low thermal conductivity, and high Seebeck coefficient.^15−17^

Among the widely studied TMDs, the compound MoTe_2_ has been in the spotlight for its polymorphic nature at room temperature with an extremely small energy difference (40 meV) between the semiconducting 2H phase (α-phase) and the metastable semi-metallic 1T′ phase (β-phase).^18−20^ In addition, MoTe_2_ is the only material among group VI that can be grown in both α- (2H) and β-(1T′) phases, and their phase change has been reported by strain,^21^ joule heating,^22^ ionic liquid gating,^23^ electrostatic doping,^24^ and laser irradiation.^18^ This makes MoTe_2_ an excellent candidate for the fabrication of two-dimensional (2D) hetero-phase homojunction and opens new possibilities for designing low-cost applications in utilizing the phase-engineering of MoTe_2_. Nevertheless, as reported earlier, the characteristics of MoTe_2_ are greatly influenced by external perturbations such as thermal stress compared with other counterpart materials.^25^ The influence of thermal stress is directly proportional to the heat capacity and thermal conductivity of a certain material. This combined with the readily phase change of MoTe_2_ by localized heating may generate Te migration during the operation of MoTe_2_-based memory devices^26^ or heat-induced structural phase transition of the 2D MoTe_2_ film under Joule heating.^27^ Understanding phonon properties would enable the extraction of the thermal coefficient and thermal conductivity, which is of fundamental importance for such device applications. The thermal transport properties of MoTe_2_ have only been investigated theoretically via computational methods^28,29^ which revealed MoTe_2_ as a promising candidate for thermoelectric applications. Although these studies provide a basic understanding of thermal transport in monolayer 2H-MoTe_2_ for future applications, the thermal transport properties have not been experimentally analyzed.

The noncontact optothermal Raman technique has been considered one of the most robust methods for measuring the thermal properties and thermal conductivity of a wide range of 2D materials.^30−38^ In addition, this technique is not limited to atomically thin layers and has also been extended to relatively thick layers with adequate laser power intensity to cause the local heating of the sample.^39,40^ In this work, we present the temperature- and power-absorbed- dependent analysis of thin MoTe_2_ films that permit the extraction and calculation of the interfacial thermal conductance with the substrate and the thermal conductivity of the material. The obtained room-temperature thermal conductivity for MoTe_2_ flake is remarkably low compared with other TMD materials characterized by the optothermal Raman method. These results provide valuable information about the understanding of the heat conduction of MoTe_2_ and should be considered in the fabrication of polymorphic engineering of MoTe_2_ for thermoelectric and optoelectronic applications.

## Results and Discussion

**Figure 1:**
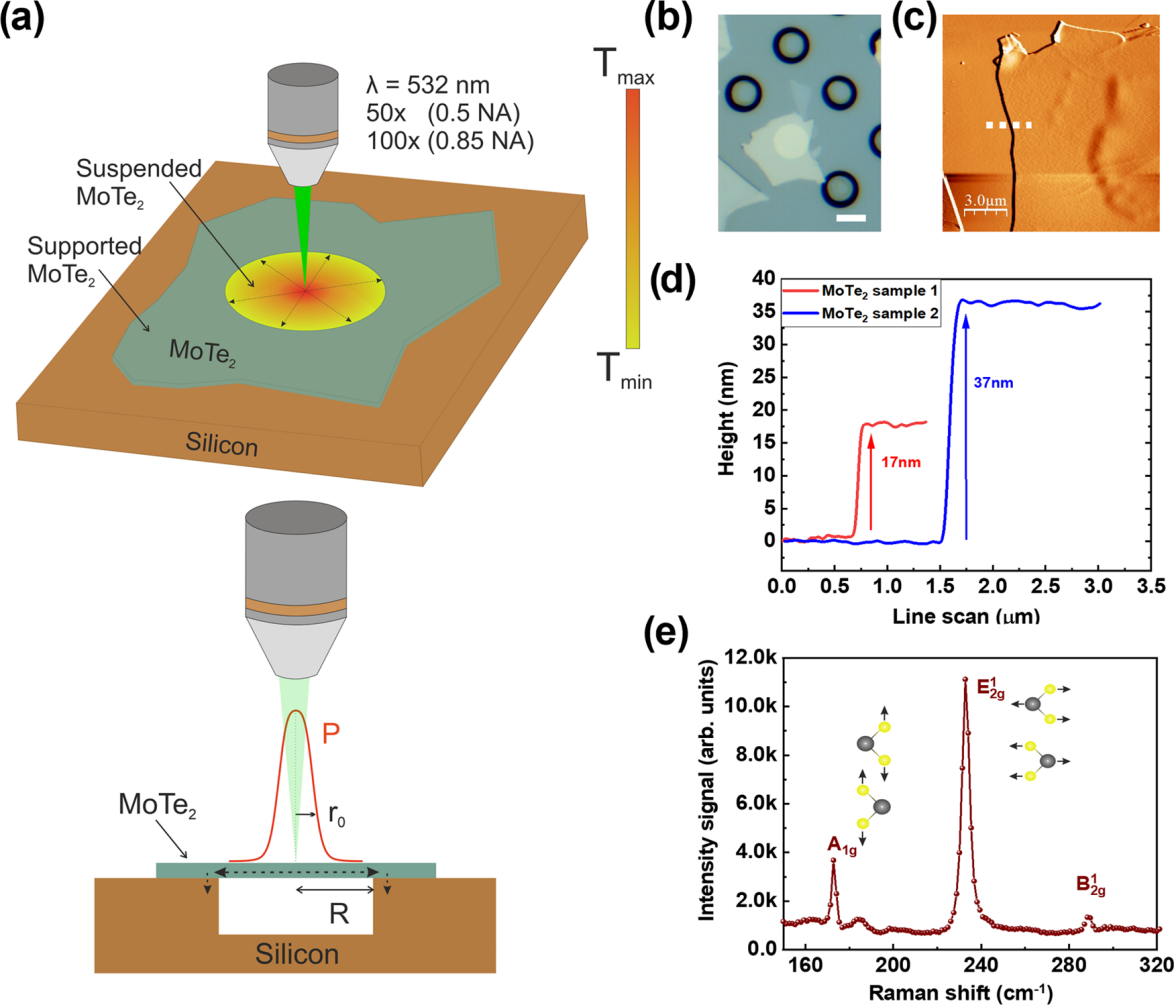
(a) Sketch of optothermal Raman experiment for the MoTe_2_ samples placed on the Si substrate. (b) Optical microscope image of MoTe_2_ sample transferred over the prefabricated holes. (c) AFM example of a 37 nm thick sample and (d) step height of the exfoliated MoTe_2_ samples after being transferred to the Si substrate. (e) Room-temperature Raman spectrum of supported MoTe_2_ (sample 1, 17 nm flake) with the schematic representation of the E_2g_^1^ and A_1g_ modes.

Prior to the detailed optothermal analysis, multilayered MoTe_2_ flakes were mechanically exfoliated from commercially purchased MoTe_2_ crystal using Scotch tape and transferred onto clean Si substrates with prefabricated holes. The Si substrate presents a higher thermal conductivity (around 150 W/m·K)^41^ that can act as a heat sink. This enables the direct comparison of the temperature and power coefficients between the suspended and the supported characterized samples. The schematic of the optothermal Raman technique employed in the characterization of MoTe_2_ flakes suspended over circular holes on the Si substrate can be observed in Figure 1a. This technique requires a two-step procedure. First, the temperature dependence of the position of the optical phonon modes of the MoTe_2_ flakes is studied to extract the first-order temperature coefficient (χ_*T*_), which acts as a Raman thermometer. After that, the laser-power-dependent Raman spectra of the selected phonon modes are recorded to determine the amount of absorbed power on either the supported or the suspended MoTe_2_ at room temperature. The 100× and 50× objectives with different numerical apertures were employed to perform the power-dependent measurements. Having the local temperature raised by the laser power, the heat diffusion equations can be solved. The cross section of the MoTe_2_ on the Si substrate is also displayed in Figure 1a, where holes with a diameter of 10 μm were fabricated by maskless lithography (see the Methods Section). The microscope image in Figure 1b presents a thin MoTe_2_ flake that covers holes on the Si substrate. The multilayer MoTe_2_ samples with a thickness in the range of 15–40 nm are preferred in our experiments due to the fact that multilayer samples are environmentally robust, exhibit band gap close to Silicon, and can be transformed from semiconducting to the metallic phase with laser. Importantly, we use two samples with similar thicknesses, noted as sample 1 (17 nm thick) and sample 2 (37 nm thick), in order to monitor the reproducibility of the employed method for the determination of thermal conductivity. The height profile measured by atomic force microscope (AFM) across the edge of the flakes is displayed in Figure 1d for both samples. To corroborate the quality of the transferred sample over the holes, the samples were characterized by micro-Raman spectroscopy at room temperature and low power intensity, as presented in Figure 1e.

As the point group of semiconducting bulk 2H-MoTe_2_ belongs to D_6h_ symmetry, the unit cell is composed of six atoms, resulting in 18 phonon modes (15 of them are optical modes). Due to the center of inversion, bulk 2H-MoTe_2_ can be split into gerade (marked with subindex g) and ungerade (subindex u) modes. The gerade modes present a change in the polarizability but do not have a dipole moment, and they are Raman-active modes. Ungerade modes have a dipole moment and, therefore, these modes are infrared active. Since bulk 2H-MoTe_2_ is a center-symmetric crystal, the Raman modes cannot appear in an infrared spectrum and vice versa, they are exclusive. Thus, the irreducible representation of the lattice vibration at Γ point of the Brillouin zone (zone center) can be expressed as Γ= A_1g_ + 2A_2u_ + 2B_2g_ + B_1u_ + 2E_1u_ + 2E_2g_ + E_2u_ (E modes are double degenerated), where the Raman-active modes are A_1g_, E_1g_, and E_2g_, while the bulk B_2g_ modes are optically inactive. In the selected region of the Raman spectrum (17 nm flake) of Figure 1e can be identified the characteristic optical phonon modes A_1g_, E_2g_^1^ and the optically silent B_2g_^1^ which begins to appear because of the lack of symmetry along the layer direction (not observable in the 37 nm flake). The E_2g_^1^ mode involves the vibration of the Mo atoms in-plane with respect to the Te atoms, which vibrate in the opposite direction. This mode is usually used as the characterization peak for thermal transport measurements due to its clear and intense signal in comparison with the other modes,^38^ while in the optical A_1g_ mode Mo vibrates out-of-plane and in in-phase directions. For the optothermal analysis, we will focus on the E_2g_^1^ and the A_1g_ modes which are optically active in both samples.

**Figure 2:**
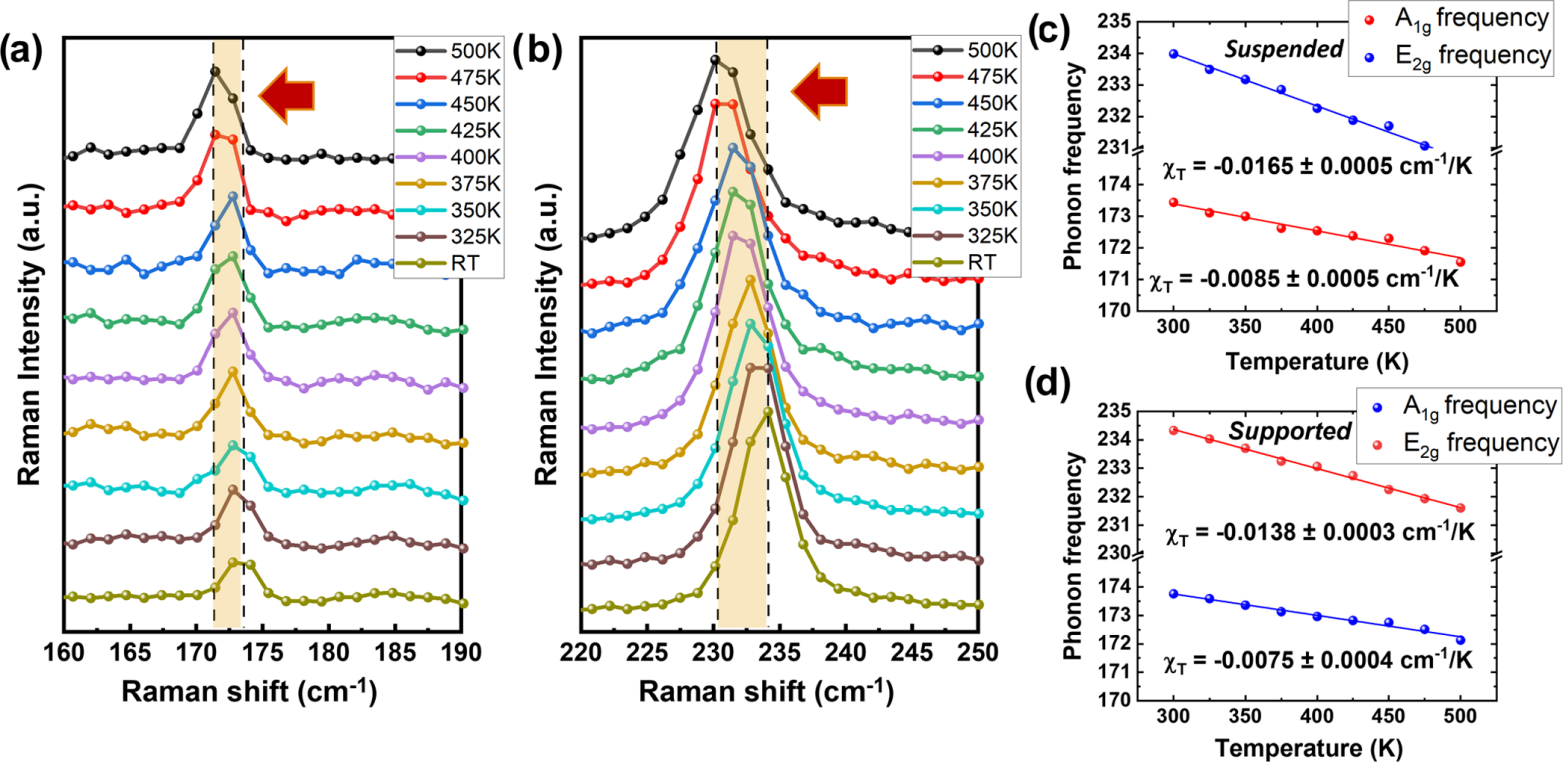
(a) Temperature-dependent Raman spectra of (a) A_1g_ and (b) E_2g_^1^ for suspended MoTe_2_ (sample 1, 17 nm) with a temperature range from room temperature (around 293 K) to 500 K. Temperature-dependent coefficients of the characterized E_2g_^1^ (blue circles) and A_1g_ (red circles) phonon modes for the (c) suspended and (d) supported MoTe_2_. These points have been individually extracted from the Lorentzian fit of the Raman peaks.

Figure 2 shows the calibration of temperature coefficients for the supported and suspended parts of the E_2g_^1^ and the A_1g_ modes while heating the substrate (temperature ranges from room temperature to 500 K). All of the measurements were collected after the sample’s temperature was stable. The temperature increase leads to softening of the active Raman modes, and the E_2g_^1^ and A_1g_ modes follow a systematic redshift with increasing temperature (see Figure 2a and b for suspended MoTe_2_ sample 1). This evolution is given approximately by the linear equation Δω = χ_*T*_Δ*T* (Figure 2c and d), where χ_*T*_ is the first-order temperature coefficient for MoTe_2_ optical modes and Δ*T* is the temperature change. Note that E_2g_^1^, the in-plane phonon mode, is more affected by the strain^42^ and thermal expansion of the lattice with respect to the A_1g_. Furthermore, the fitting χ_*T*_ parameters for suspended MoTe_2_ are slightly different from the supported part because of the influence of the thermal expansion coefficient mismatch between the sample and the substrate.^43^

**Figure 3:**
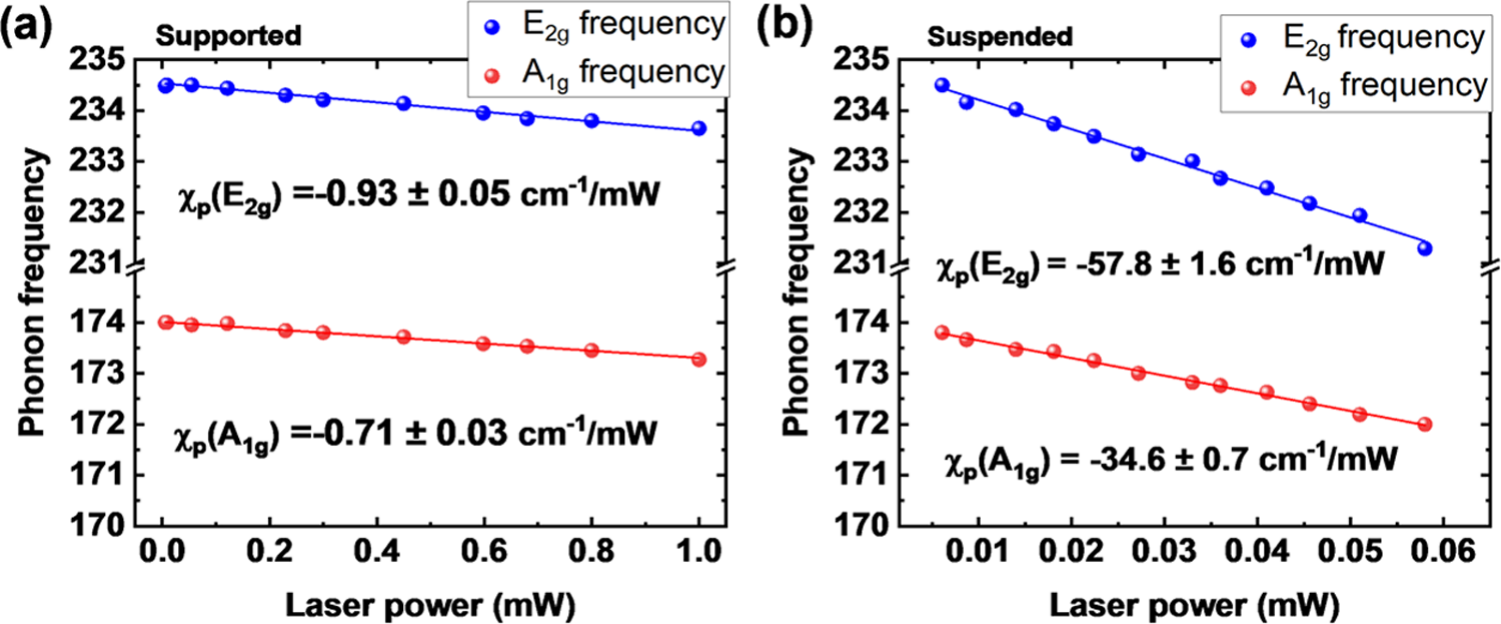
Power dependence of E_2g_^1^ (blue circles) and A_1g_ (red circles) peak frequencies of (a) supported and (b) suspended MoTe_2_ using 100× objective, including the respective χ_*P*_ values.

To extract the linear relation between the sample temperature and the absorbed laser power, we investigated the effect of laser-induced heat on the selected modes for MoTe_2_ suspended and over Si substrate at various laser powers. All spectra were collected using 50× (numerical aperture, NA = 0.5) and 100× (NA = 0.85) objectives. To avoid sample heating and the appearance of nonlinear effects, the Raman characterization was done at lower power intensities within the linear dependence range (see Figure S1, Supporting Information). For example, in Figure 3, we can observe the power-dependence evolution of the mentioned modes for supported (Figure 3a) and suspended (Figure 3b) MoTe_2_ sample 1 using 100× objective. The data has been extracted from the Lorentzian fit of the phonon modes as a function of the laser power. The power-dependence measurements for the MoTe_2_ sample 2 and Raman shift evolution using a 50× objective can be found in Figures S4 and S5. In general, we have observed that in all of the cases, the Raman peaks downshift as the laser power increases, indicating a local heating of the sample. The redshift of E_2g_^1^ and the A_1g_ modes is more evident for the suspended samples because the incident laser beam is the main contributor to the heat change of the sample.^32^ The linear Raman shift rates can be expressed by the equation Δω = χ_*P*_Δ*P*, where χ_*P*_ is the first-order power coefficient and Δ*P* is the change in the laser power.

By combining equations for χ_*T*_ and χ_*P*_, we can obtain the change in temperature as a function of change in the absorbed laser power, defined as the thermal resistance *R*_m_
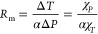
1where α is the absorption. We assume an absorption coefficient of 4.69 × 10^7^ 1/m for MoTe_2_,^44^ which gives α values of 0.55 and 0.82 for 17 and 37 nm MoTe_2_ samples, respectively.

**Figure 4:**
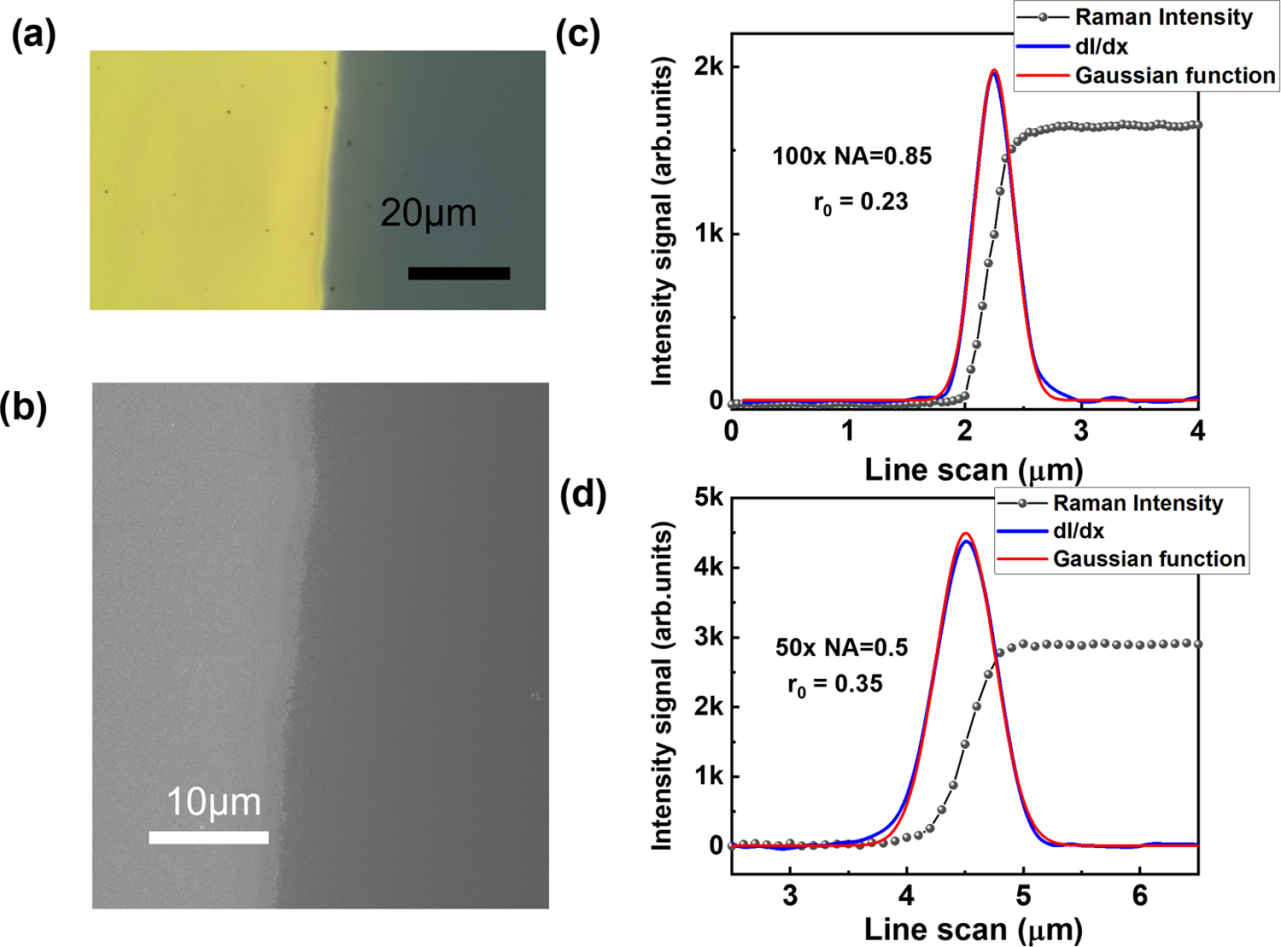
(a) Optical and (b) scanning electron microscope images of the Au edge. For the measurement of the beam spot profile, the cleaned Si substrate was coated with a layer of Au (about 300 nm thick) using an electron-beam evaporator. The dotted line corresponds to the scan line of the beam profile and indicates the extracted Raman intensity of the laser spot from (c) 100× objective (NA = 0.85) and (d) 50× (NA = 0.5).

Thermal resistance given by eq 1 correlates the value of the temperature increase with increasing laser power. To link this value to the thermal conductivity of the material, we need to calculate the theoretically predicted temperature profile in the case of an external heat source applied to the material, which in our case is the illuminating laser beam. The heat is diffused in the plane of the MoTe_2_, and the efficiency of the diffusion, and thus the overall temperature increase, is directly related to the thermal conductivity. Also, in the supported parts of the MoTe_2_, the heat is transferred to the Si substrate, which acts as a heat sink. Neglecting the heat convection to air and assuming continuous heating by the laser beam, the steady-state temperature profile is determined by the heat diffusion equations^30^
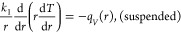
2
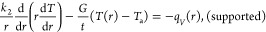
3where *k*_1(2)_ is the thermal conductivity of the suspended (supported) MoTe_2_, *G* is the interfacial thermal conductance between the MoTe_2_ and the substrate, *t* is the thickness of the MoTe_2_ flake, *T*_a_ is the ambient temperature, and *q*_*V*_ is the volumetric optical heating from the Gaussian laser beam
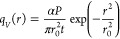
4where *r*_0_ is the laser beam radius. The laser beam radius was experimentally determined by performing line scan Raman spectroscopy across an Au layer-coated cleavage edge (Figure 4a,b) using 100× and 50× objectives. Then, the Si peak intensity was extracted as a function of the moving position (d*I*/d*x*), which can be fitted by a Gaussian function exp(−*x*^2^/*r*_0_^2^) for both: 100× (Figure 4c) and 50× (Figure 4d). The experimental values of *r*_0_ were 0.23 and 0.35 μm, which slightly differs from the estimated through numerical aperture 0.19 and 0.33 μm for 100× (NA = 0.85) and 50× (NA = 0.5), respectively. The temperature profile depends on whether the MoTe_2_ layer is either suspended or supported, leading to different boundary conditions. In all cases, the applied boundary conditions are d*T*/d*r*|_*r*=0_ = 0, and *T*(*r* → ∞) = *T*(*a*), ensuring that the temperature stays finite at the origin and that the temperature is the ambient temperature far away from the laser-illuminated area.

We will first start our analysis on supported MoTe_2_, for which eq 3 applies everywhere. This analysis will help us obtain *k*_2_ and *G*, which are also needed to calculate the thermal conductivity for the suspended MoTe_2_, *k*_1_. By applying the aforementioned boundary conditions, we can solve the temperature profile by using the variation of parameters technique^30,45^
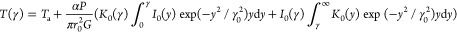
5where we have defined an auxiliary parameter  and γ_0_ = γ(*r*_0_). The area of the illuminated laser beam determines the experimentally observed temperature *T*_m_. This is obtained as an average temperature over the 2D space weighed by the Gaussian beam profile
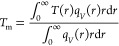
6and thus the observed temperature change is Δ*T* = *T*_m_ – *T*_a_, and by dividing this with absorbed laser power α*P* we have the analytical value for the thermal resistance *R*_m_. Thus, by using eq 6 with 1, we have linked the experimentally obtained temperature increase with the analytical temperature profile determined by the thermal conductivity *k*_2_ and interfacial thermal conductance *G*, which are the two unknown values. Thus, two different experiments are needed to determine them uniquely.

To determine *k*_2_ and *G*, we follow the method presented by Cai et al.,^30^ where the power-dependent Raman measurements are done for two different objectives, 100× and 50× in our case, which give two different thermal resistances *R*_m_. By taking the ratio of these two values, we get a formula solely dependent on the ratio of the two parameters, *k*_2_/*G*, which can then be solved numerically. Then, we applied the solved ratio to the thermal resistance of one of the objectives to extract both *k*_2_ and *G*. The results for the characterized modes of samples 1 and 2 are summarized in Table 1.

**Table 1:** Temperature and Power Coefficients, Thermal Conductivities, and Interfacial Thermal Conductances of MoTe_2_

			χ_*P*_ [cm^–1^/mW]			
MoTe_2_	mode	χ_*T*_ [cm^–1^/K]	100×	50×	*G* [MW/m^2^K]	*k* [W/m·K]
sample 1	A_1g_	–0.0085 ± 0.0006	–34.6 ± 0.7	–21.4 ± 0.7		3.72 ± 0.26
suspended	E_2g_^1^	–0.0165 ± 0.0005	–57.8 ± 1.6	–32.3 ± 1.3		5.16 ± 0.24
sample 1	A_1g_	–0.0075 ± 0.0004	–0.71 ± 0.03	–0.32 ± 0.02	∼16.2^a^	∼2.35^a^
supported	E_2g_^1^	–0.0138 ± 0.0003	–0.93 ± 0.05	–0.41 ± 0.01	∼23.9^a^	∼0.80^a^
sample 2	A_1g_	–0.00977 ± 0.0004	–8.1 ± 0.6	–10.0 ± 0.6		13.2 ± 1.2
suspended	E_2g_^1^	–0.01519 ± 0.0007	–12.9 ± 1.2	–13.5 ± 0.9		12.8 ± 1.4
sample 2	A_1g_	–0.0075 ± 0.0007	–1.05 ± 0.03	–0.67 ± 0.03	5.5 ± 1.7	18.1 ± 6.4
supported	E_2g_^1^	–0.01402 ± 0.0003	–1.35 ± 0.03	–0.83 ± 0.05	9.7 ± 2.3	22.0 ± 9.3

a The extracted values of the supported sample 1 require further experimental analysis with different substrates for better estimation of the errors. More information can be found in Figure S6.

When the sample is suspended over the holes, eq 2 applies in the region 0 ≤ *r* < *R* and eq 3 in *r* ≥ *R*, where *R* is the radius of the hole. In this case, two additional boundary conditions are needed, namely, that the temperature profile is continuous at *R*, and −2π*Rtk*_1,2_d*T*/d*r*|_*r* = *R*_ = α*P*, indicating that the heat transferred out of the suspended flake must equal the absorbed laser power.

By solving the differential equation, and assuming that *r*_0_ ≪ *R*, we get the temperature profile for suspended MoTe_2_, namely, in the region 0 ≤ *r* < *R*
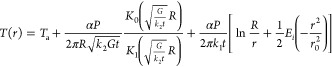
7where *E_i_*(−*r*^2^/*r*_0_^2^) is the exponential integral.^46^ The temperature profile in the region *r* ≥ *R* is given by eq (S1) in the Supplementary Information.

We can obtain the formula for *k*_1_ by using eq 6 in 7, and then implementation of the power-dependent Raman measurement only for one objective would be sufficient, as there is only one unknown parameter. By carrying out the integration and then solving for *k*_1_, we get the formula
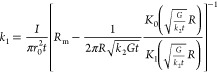
8where .

**Figure 5:**
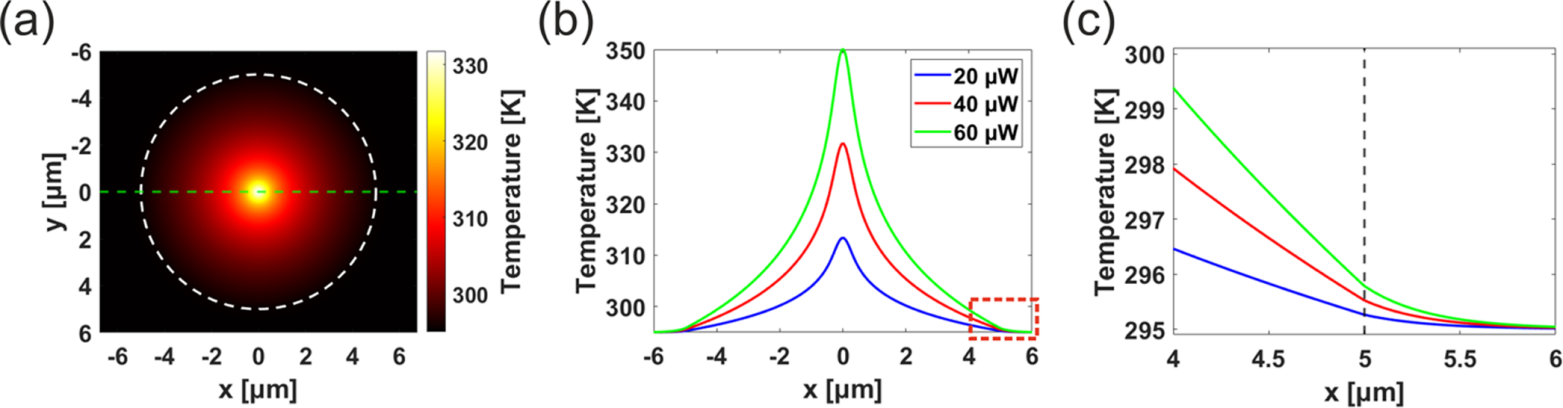
Temperature profile for suspended 37 nm MoTe_2_ illuminated by a laser beam with 100× objective. (a) Heat map of the temperature profile for 40 μW laser power with a dashed white line indicating the edge of the hole. (b) Temperature profile along the x-axis indicated by the green dashed line in (a) for different laser powers. (c) Zoomed in at the edge around *R* = 5.0 μm.

The simulated temperature profiles for suspended 37 nm MoTe_2_ are given in Figure 5, with 100× objective. Figure 5a shows the temperature profile over the 2D plane for 40 μW laser power, with Figure 5b limiting itself to the x-axis, indicated by the green dashed line in Figure 5a. Figure 5c shows the zoomed-in part near the edges of the Si holes, where the material changes from suspended to supported, and the temperature decays to the room temperature *T*_a_ = 295 K. The decay length of the temperature on the supported part of 2D material is determined by the thermal conductivity of the material and the interfacial thermal conductance between the material and the substrate. Still, overall the temperature of the sample is close to the room temperature at the edges due to the large diameter of the holes compared with the spot size of the Gaussian beam and the Si substrate working as an effective heat sink. Thus, the exact values of *k*_2_ and *G* do not significantly affect the calculated value of *k*_1_, and the results are not substantially different if the substrate is assumed to be a perfect heat sink.^40^ However, this assumption would not be valid in the case of much smaller holes or less heat-conducting substrate and better conducting 2D material.^30,33^ Hence, we used the more comprehensive method by analyzing the supported part of the MoTe_2_, which validates our results and gives more insight into the thermal dynamics of the material.

From the Raman measurements on the suspended 17 nm MoTe_2_, we obtain χ_*T*_ = −0.0085 ± 0.0006 cm^–1^/*K* and χ_*P*_ = −34.6 ± 0.8 cm^–1^/mW for the A_1g_ mode and 100× objective, giving us thermal resistance *R*_m_ = 7.3 × 10^6^ K/W. Inserting this into eq 8, the thermal conductivity is extracted as *k*_1_ = 3.72 ± 0.26 W/m·K, which is substantially smaller when compared with other 2D materials. The reason might be high strain due to mechanical exfoliation, which can cause a significant decrease in thermal conductivity. Repeating the same analysis for 37 nm MoTe_2_, we obtain *k*_1_ = 13.2 ± 1.2 W/m·K.

Table 1 summarizes the experimental results and the calculated thermal conductivities for supported and suspended MoTe_2_ for both 17 nm and 37 nm samples. The extracted thermal conductivities *k*_2_ = 18.1 ± 6.4 W/m·K (supported) and *k*_1_ = 13.2 ± 1.2 W/m·K (suspended) for 37 nm MoTe_2_ are considerably higher than the corresponding values for the 17 nm sample. This is in agreement with the results presented for MoS_2_ by Yuan et al.,^47^ where nonmonotonic thickness dependence of thermal conductivity was observed, and in particular, 17 nm thickness is expected to have lower thermal conductivity than relatively thicker samples due to more considerable surface scattering. The differences in thermal conductivity can also be attributed to the results of mechanical exfoliation, which are expected to vary from sample to sample. Furthermore, the interfacial thermal conductance between the MoTe_2_ and Si substrate is lower for the 37 nm sample (*G* = 5.6 ± 1.7 MW/m^2^K) than for the 17 nm sample (*G* = 16.2 MW/m^2^K) in contrast to previous analysis performed on MoS_2_.^33^ This can be explained by the differences during the dry transfer process, as the thinner sample required an additional heating/cooling step resulting in a significantly improved contact between the substrate and the sample (see the Methods Section). Nevertheless, further analysis of the interfacial thermal conductance with different substrates and different MoTe_2_ thicknesses are required. analysis.

## Conclusions

In summary, we used the optothermal Raman method to experimentally determine the in-plane thermal conductivities and interfacial thermal conductance of MoTe_2_ thin films with thicknesses 17 and 37 nm. The laser spot sizes were experimentally obtained by the knife edge method, which was slightly different than the theoretically predicted values and was proved to be essential to extract accurate thermal conductivity for the supported MoTe_2_. The thermal conductivity of MoTe_2_ was shown to be considerably lower than that of other TMD materials, with 17 nm MoTe_2_ having remarkably low thermal conductivities *k*_1_ = 3.72 ± 0.26 W/m·K and *k*_2_ = 2.35 W/m·K for the suspended and supported parts, respectively. Overall, we performed the first experimental study of the thermal properties in suspended MoTe_2_ using the optothermal Raman technique. Our experimental results provide essential input on the fundamental thermal parameters and heat dissipation of MoTe_2_ and confirm this material as a potential candidate for thermoelectric applications.

## Methods

### Sample Fabrication

The characterized MoTe_2_ flakes were first mechanically exfoliated from clean bulk crystals (HQ Graphene, 2H phase) using the polydimethylsiloxane (PDMS)-assisted dry transfer method.^48^ The samples were transferred onto clean Si substrates with diameter holes of 9–10 μm and depth around 3 μm. The large diameter and depth of the fabricated holes ensure the elimination of possible artifacts from the influence of the substrate for the extraction of thermal conductivity. For the transfer, the positions of the Si holes and the MoTe_2_ were aligned under an optical microscope (HQ Graphene, Manual transfer system HQ2D MAN). Once they were in contact, the Si substrate and PDMS were carefully separated by moving the substrate stage. The thinner sample 1 required an additional step where the sample was heated at 60 °C so that the PDMS was softened and the MoTe_2_ adhesion to the PDMS was lost. However, the thermal expansion coefficient of both materials can lead to the formation of wrinkles which may affect the thermal conductivity and interfacial thermal conductance. To preserve the quality of the material, we kept the samples in a dry nitrogen box.

### Fabrication of Si Holes

The silicon samples were cleaned using acetone, isopropanol, and O_2_ plasma. The prepatterned holes of the Si substrates were fabricated before the deposition of the MoTe_2_ as follows: (a) Photoresist AZ ECI 3027 was spin-coated on the samples at 3000 rpm and baked for 60 s at 100 °C. (b) The resist was exposed for 160 ms with Heidelberg μPG 501 maskless lithography system. (c) Samples were developed in 1 min 30 s in AZ-726 MIF developer. (d) After that, silicon was etched with reactive ion etching (RIE) for 8 min. (e) RIE was done with SF6 and O_2_ plasma (300 W, 50 mTorr, and 6 sccm for O_2_ and 7 sccm for SF6). (f) Lastly, the remaining resist was removed with a Microposit 1165 remover.

### Raman Measurement

Raman spectra were collected using an inVia Qontor confocal Raman spectroscope (Renishaw) under 532 nm laser line excitation. The laser beam focused on the sample with 100× (NA = 0.85) and 50× (NA = 0.5) microscope objectives. All measurements were calibrated with silicon samples using the characteristic phonon peaks at 520.5 cm^–1^. After measuring the phonon for calibration, we kept the spectrometer in the corresponding position in order to avoid displacements in the spectra; thus, our experimental values have an uncertainty smaller than 0.2 cm^–1^. The samples were placed on a temperature-controlled heating platform to measure Raman peak shifts with temperature and laser power change (Linkam Stage THMS600). The samples were left to thermalize for 20 min at each temperature. To prevent additional laser heating, the temperature-dependent measurements were performed at low power intensity and close to the minimum value of the linear region presented in the power-dependent measurements for each sample (see Figures 3, S1, S4, and S5).

## Data Availability

The authors declare that the data supporting the findings of this study are available within the paper and in the Supporting Information files.

## Supporting Information Available

The online version contains supplementary material and includes nonlinear region of the absorbed power, laser irradiation in a few-layer MoTe_2_, temperature- and power-dependent measurements for suspended and supported, estimation of the error of the thermal properties, the temperature profile of sample 1, and AFM of sample 1 (PDF)
