# Supplementary material for: Unraveling Thermal Transport Properties of MoTe2 Thin Films Using the Optothermal Raman Technique
Source: ACS Appl Mater Interfaces. 2023 Jul 12;15(29):35692–700. doi: 10.1021/acsami.3c06134 (PMC10375478; doi:10.1021/acsami.3c06134)
Supplement: Supplementary file 1 — am3c06134_si_001.pdf [file am3c06134_si_001.pdf]

Supporting information: Unraveling thermal transport properties of $MoTe_2$ thin films using the optothermal Raman technique

Carlos Rodriguez-Fernandez,[*,†] Arttu Nieminen,[†] Faisal Ahmed,[‡] Jesse Pietila,[†] Harri Lipsanen,[‡] and Humeyra Caglayan[*,†]

†*Faculty of Engineering and Natural Sciences, Photonics, Tampere University, 33720 Tampere, Finland*

‡*Department of Electronics and Nanoengineering, Aalto University, P.O. Box 13500, FI-00076 Aalto, Finland.*

E-mail: carlos.rodriguezfernandez@tuni.fi; humeyra.caglayan@tuni.fi

Nonlinearity of the power absorbed and phase change of the supported $MoTe_2$

Power-dependent measurements of the main text have been performed considering the linear region of the laser power absorbed (see Figure S1a). This linear evolution of the sample differs whether the sample is suspended or supported, as well as the thickness of the flake. For example, by using a 100× objective in the supported part of sample 1 (17 nm), the nonlinearity of the Raman modes occurs above 1 mW of the laser power. Then the observed redshift of E^1_{2g} (blue circles) and A_{1g} (red circles) saturates, and the trigonal 2H phase is distorted into the octahedral 1T' structure (Figure S1b). As a result of the absorbed laser power, the E^1_{2g} and A_{1g} disappear, and two new features emerge in the Raman spectra at 126 and 140 cm^{-1} that corresponds to the modes of the induced 1T'-$MoTe_2$. For the purpose of comparison, the phase change by laser irradiation has been performed in a different sample with similar thickness.

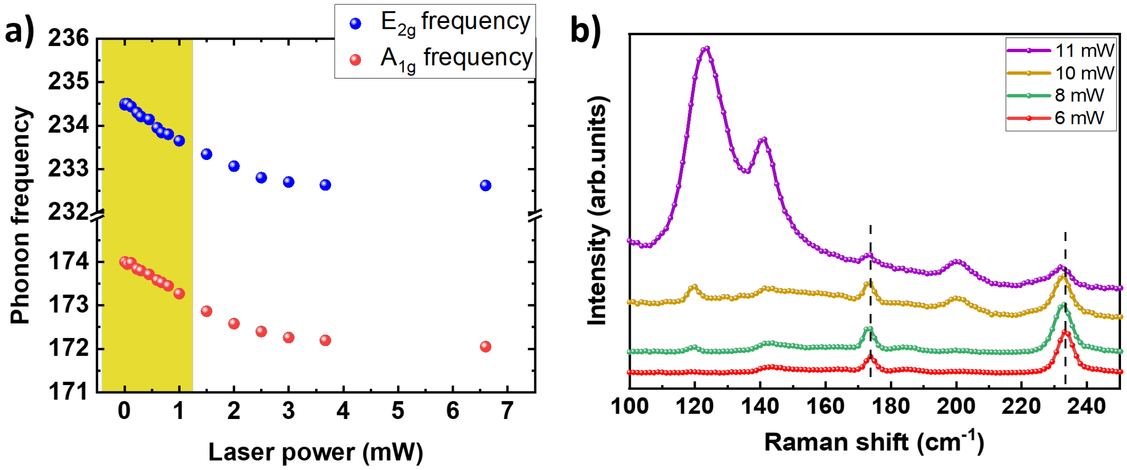

Figure S1: a) Power dependent measurements of the supported sample 1 of the E^1_{2g} and A_{1g} for laser power up to 6.6 mW b)Beyond 6 mW the lattice is deformed, and the 1T' is induced by laser irradiation.

Phase change by laser irradiation below 10-15 nm of thickness

The selected samples' thicknesses for the optothermal analysis have been selected because those are more robust to ambient perturbations and can be considered in the polymorphic engineering of $MoTe_2$. In our experiments, samples with thicknesses below 10-15 nm thick cannot be laser-driven from the 2H to 1T' phase since most of the material is evaporated and they are environmentally unstable using the optothermal Raman technique.

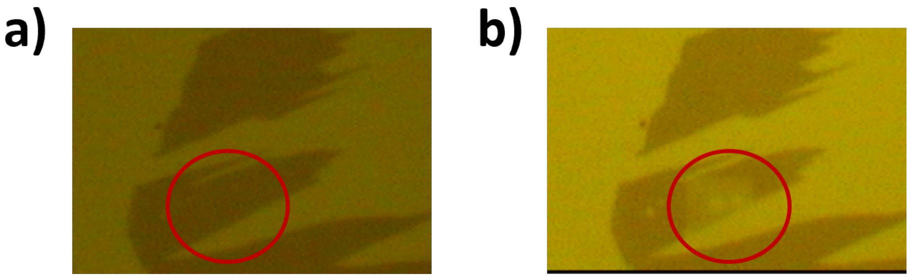

Figure S2: Optical microscope image of few-layer $MoTe_2$ before a) laser irradiation and b) after laser irradiation.

Temperature dependence measurements of sample 2

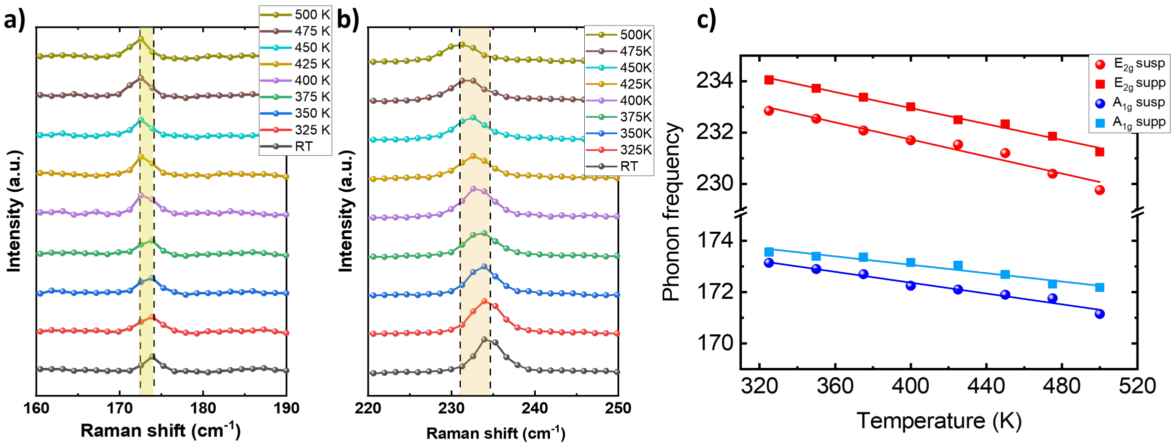

Figure S3: a) Temperature-dependent Raman spectra of a) A_{1g} and b) E^1_{2g} for supported $MoTe_2$ (sample 2, 37nm) with temperature range from room temperature to 500 K. c) Temperature-dependent Raman peak positions of the characterized E^1_{2g} (red square are related to the supported and red circles to the suspended) and A_{1g} (blue square are related to the supported and blue circles to the suspended) phonon modes for the sample 2 $MoTe_2$. All the points have been individually extracted from the Lorentzian fit of the Raman peaks.

Power dependent measurements

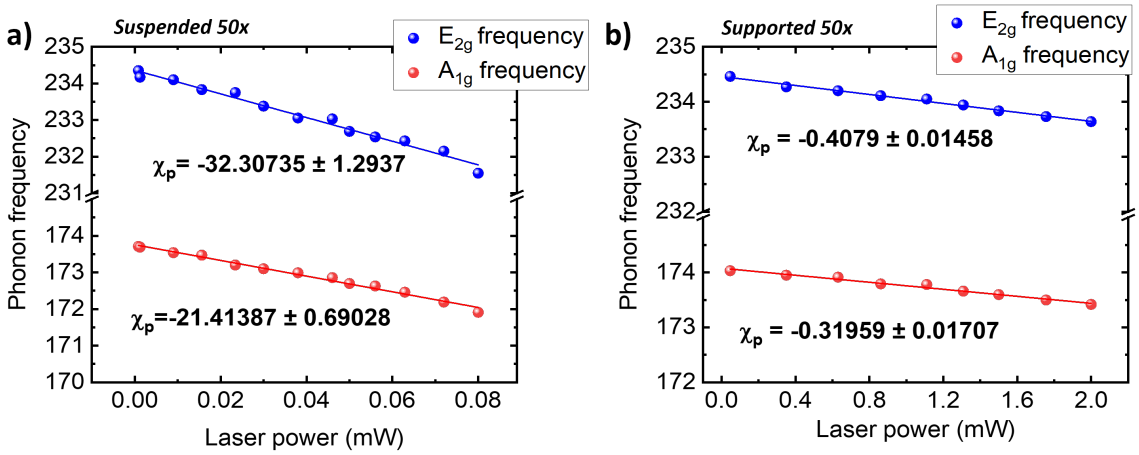

Figure S4: A_{1g} and E^1_{2g} phonon mode frequency of sample 1 (17 nm) for a) suspended and b) supported $MoTe_2$ as a function of absorbed laser power using 50× objective with the linear fit.

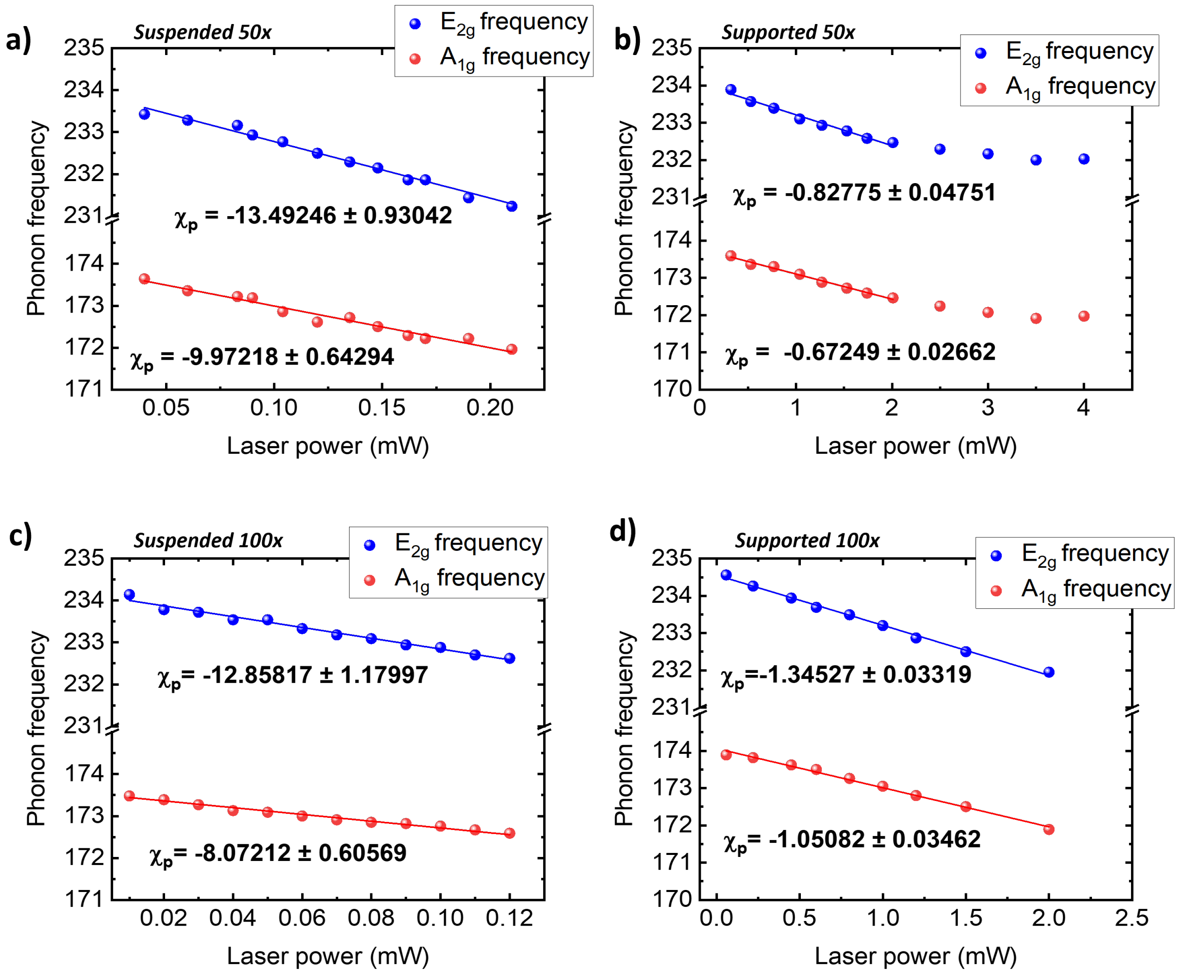

Figure S5: A_{1g} and E^1_{2g} phonon mode frequency of sample 2 (37 nm) for suspended and supported $MoTe_2$ as a function of absorbed laser power using a-b) 50× and c-d) 100× objective with the linear fit.

Error estimation of the supported part

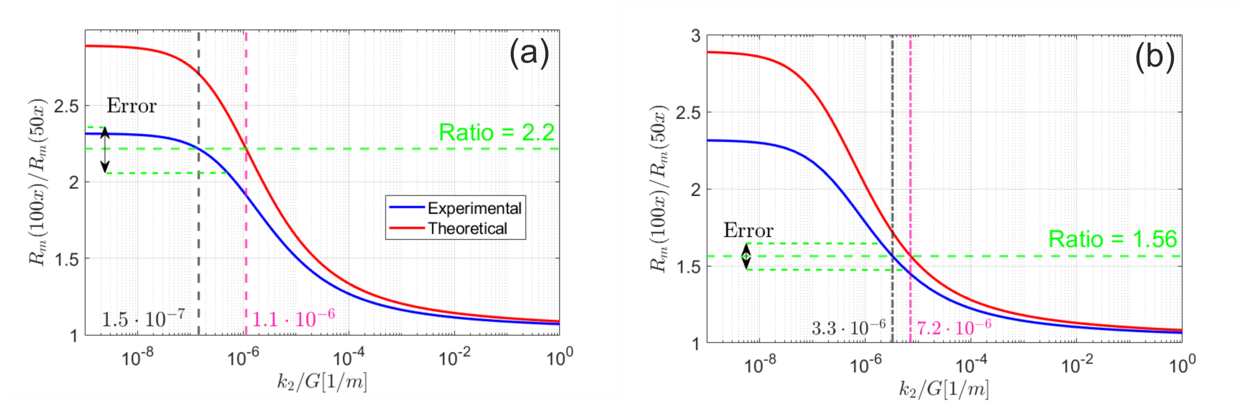

Figure S6: Theoretically predicted ratios of the thermal resistances for 100× and 50× objectives $R_m(100\times)/R_m(50\times)$ as a function of the ratio k_2/G. The red curves present the results based on the theoretical spot sizes from the numerical aperture of the objectives, while the blue curves show the results based on the experimentally determined spot sizes. The green dashed lines indicate the experimentally obtained ratios and their uncertainties, using the A_{1g} mode values. The points where the curves intersect give the solution to k_2/G. (a) Thermal resistance ratio curves for the 17 nm sample. (b) The corresponding curve for the 37 nm sample.

Figure S6 presents the theoretically predicted ratios of the thermal resistances for 100× and 50× objectives $R_m(100\times)/R_m(50\times)$ as a function of the ratio k_2/G, where the red line is the obtained curve when using the theoretically predicted values of the beam spot sizes, and the blue line corresponds to the experimentally determined spot sizes. The green dashed line indicates the experimental value of the ratio of thermal resistances, with errors indicated by the black arrows. The solutions for k_2/G are given as points where the green line intersects with the blue or red curves. Figure S6 (a) presents the thermal resistance for the 17 nm sample. The solution given by the experimental and theoretical spot sizes differ by one order of magnitude; thus, determining the actual spot sizes of the heating laser beam is essential to get reliable results with this method. The upper limit of the experimental uncertainty of the $R_m(100\times)/R_m(50\times)$ is on the region where no solution for the k_2/G is obtained due to the Si substrate having too big interfacial thermal conductance with the $MoTe_2$. One option to decrease the uncertainties of the thermal properties would be to transfer$MoTe_2$

onto different substrates with lower thermal conductivities, such as silica. In general, the interfacial thermal conductance analysis with distinctive substrates would contribute to a better understanding of thermal management. Figure S6 (b) gives the corresponding curves for the 37 nm sample. The difference of k_2/G between the theoretical and experimental spot sizes is substantially smaller. The uncertainties in the experimentally obtained ratio cause much smaller uncertainty in the obtained k_2/G, as the position on the curve is at a much steeper part in contrast to the case for the 17 nm sample, where the solution is on the saturated part of the curve. However, the uncertainties are still quite large due to the logarithmic scale of the x-axis, which means that the theoretically obtained ratio is really sensitive to the experimental value of the thermal resistances R_m.

Temperature profile of sample 1

In the case of suspended $MoTe_2$, the sample is supported in the region $r \geq R$, in which Eq. (3) from the main text applies, with proper boundary conditions to conform with the temperature profile of the suspended part. The temperature profile for the supported part, in this case, is

$$T(r) = T_a + \frac{\alpha P}{2\pi R\sqrt{k_2 G t}} \frac{K_0\left(\sqrt{\frac{G}{k_2 t}} r\right)}{K_1\left(\sqrt{\frac{G}{k_2 t}} R\right)}, \quad r \geq R. \tag{S1}$$

This temperature profile is a decaying function with the limit $T(r \to \infty) = T_a$, and G and k_2 determine the decay length. In the case of $MoTe_2$, the low thermal conductivity k_2 and large interfacial thermal conductance G mean that the decay length is really short, and the temperature has decayed to the room temperature at $r = 4.8$ μm, namely 0.3 μm from the edge, for 17 nm $MoTe_2$. In the case of 37 nm $MoTe_2$, the temperature has decayed to room temperature at $r = 6$ μm, 1 μm from the edge, due to lower G and higher k_2 compared to 17 nm $MoTe_2$. For both samples, the temperature at $r = R$ is quite close to the room temperature due to the high interfacial thermal conductance between Si and $MoTe_2$. However, if the substrate is a low thermal conducting material, such as silica, the temperature at the edges is expected to be higher.

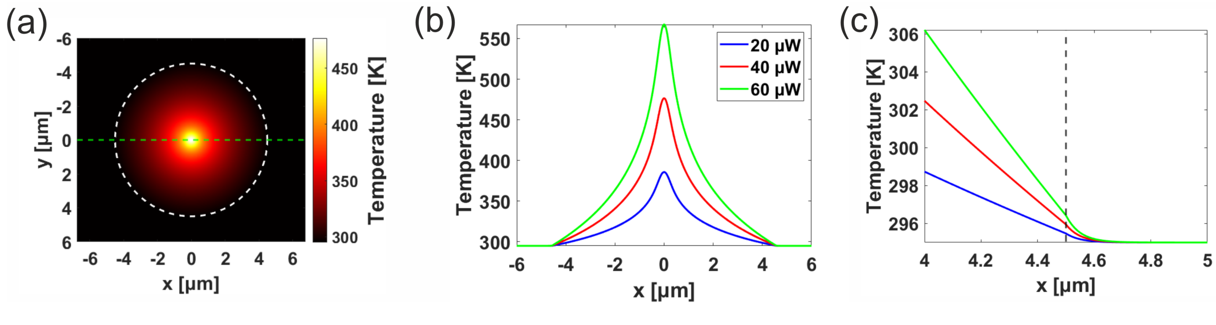

Figure S7: Temperature profile for suspended 17 nm $MoTe_2$ illuminated by a laser beam with 100× objective. (a) Heat map of the temperature profile for 40 μW laser power, with a dashed white line indicating the edge of the hole. (b) Temperature profile along the x-axis indicated by the green dashed line in (a) for different laser powers. (c) Zoomed in at the edge around $R = 4.5$ μm.

Figure S7 presents the simulated temperature profiles for the 17 nm (sample 1) when

illuminated by a laser beam with 100× objective. Figure S7 a) shows the heat map for 40 μW laser power, and b) gives the temperature profile along the x-axis for various laser powers. c) Zoomed-in part around the edge of the Si hole.

AFM image of sample 1

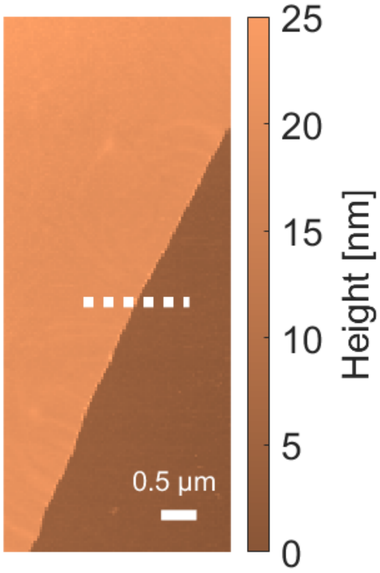

Figure S8: AFM of 17 nm thick sample. The dash-line corresponds step height of Figure 1d.
